# Termites shape their collective behavioural response based on stage of infection

**DOI:** 10.1038/s41598-018-32721-7

**Published:** 2018-09-26

**Authors:** Hannah E. Davis, Stefania Meconcelli, Renate Radek, Dino P. McMahon

**Affiliations:** 1 Institut für Biologie, Freie Universität Berlin, Königin-Luise-Str. 1-3, 14195 Berlin, Germany; 2 Department for Materials and the Environment, BAM Federal Institute for Materials Research and Testing, Unter den Eichen 87, 12205 Berlin, Germany

## Abstract

Social insects employ a range of behaviours to protect their colonies against disease, but little is known about how such collective behaviours are orchestrated. This is especially true for the social Blattodea (termites). We developed an experimental approach that allowed us to explore how the social response to disease is co-ordinated by multistep host-pathogen interactions. We infected the eastern subterranean termite *Reticulitermes flavipes* with the entomopathogenic fungus *Metarhizium anisopliae*, and then, at different stages of infection, reintroduced them to healthy nestmates and recorded behavioural responses. As expected, termites groomed pathogen-exposed individuals significantly more than controls; however, grooming was significantly elevated after fungal germination than before, demonstrating the importance of fungal status to hygienic behaviour. Significantly, we found that cannibalism became prevalent only after exposed termites became visibly ill, highlighting the importance of host condition as a cue for social hygienic behaviour. Our study reveals the presence of a coordinated social response to disease that depends on stage of infection. Specifically, we show how the host may play a key role in triggering its own sacrifice. Sacrificial self-flagging has been observed in other social insects: our results demonstrate that termites have independently evolved to both recognize and destructively respond to sickness.

## Introduction

Social insects have evolved collective behaviours to protect their colonies from disease. These social immune defences, which include pathogen avoidance, prophylactic secretions, grooming, and corpse disposal, act to protect the colony as a whole, at times at the expense of individual members^1,2^. In this latter case, sick colony members are often identified and killed, presumably to prevent the spread of disease^1,3,4^. Regulation is therefore essential, both to prevent unnecessary killing and to allow the colony to dynamically adjust its investment in other defences^1^.

Of all the social insects, the social Hymenoptera are the most well-studied. In honeybees (*Apis* spp. Linnaeus) activation of the physiological immune system by an infection results in a changed cuticular hydrocarbon profile^5^, which can then trigger the removal of the infected bee by other members of the hive^6^. Likewise, workers respond to volatiles emitted by sick or injured brood by removing them from the hive^7,8^, and factors external to the host, such as the odour of a parasite or pathogen inside a brood cell^9^, can also play a role. In ants, the situation is similar: invasive garden ant (*Lasius neglectus* Van Loon, Boomsma & Andrásfalvy) workers groom fungus-exposed pupae to prevent disease, but kill them if alerted to an internal infection by a change in cuticular hydrocarbons^3^. European fire ant (*Myrmica rubra* (Linnaeus)) workers also behave more aggressively toward fungus-infected adult nestmates once internal proliferation has begun^10^.

Comparatively little is known about how termites (Blattodea: infraorder Isoptera) shape their social immune response based on the stage of infection encountered. There is broad consensus that the initial response to a pathogen-exposed nestmate is dominated by intense allogrooming^11–15^, and cannibalism becomes more prevalent at some later stage^4,16–20^; however, when the switch occurs remains unclear. It has been observed that termites are often eaten when “moribund but not yet dead”^4^, but no study to date has attempted to identify the stage of infection at which the risk of cannibalism first begins to increase. Should a termite die from an infection, or for any other reason, necromones attract worker termites to the corpse, which they preferentially eat (necrophagy)^21^. Corpses that are too old^22^ or too numerous to consume^23^ are defecated on and then buried, isolating them from the colony^19^. Burial of live individuals has also been observed^13,24^.

Each component of the social immune response serves to prevent a pathogen from reaching the next stage in its life cycle, and ultimately to prevent an epizootic, but it will only be effective if deployed at the appropriate time. In the specific case of *Metarhizium anisopliae* (Metchnikoff) Sorokin (Ascomycota: Hypocreales), a generalist entomopathogenic fungus, allogrooming is highly effective in removing most infectious conidia from the cuticle before they can germinate^14,15,25,26^. Groomers can safely swallow the conidia^15,27^, and low-level infections acquired through contact with an infected nestmate may even boost individual anti-fungal defences^12^. Once an internal infection has been established, however, allogrooming is no longer effective^3^. The infected termite cannot be saved, and the longer it is left alive, the higher the colony-level fitness cost: a sick termite cannot work as effectively, any food that it eats will support fungal growth, and if the fungus is able to sporulate from its corpse, the whole colony will be at risk^28^.

We would therefore expect to see a switch from a grooming-dominated collective immune response to a cannibalism- and/or burial-dominated response beginning at the earliest point at which termites can detect a terminal infection. To address this, we used the eastern subterranean termite *Reticulitermes flavipes* (Kollar) and the entomopathogenic fungus *M. anisopliae* to examine how the stage of infection encountered by a colony determines the collective response. Our hypotheses were that (i) allogrooming would be most intense before conidial germination; and (ii) the shift to cannibalism would begin shortly after conidial germination. Contrary to expectations, we found that levels of grooming rose significantly after conidial germination, and that cannibalistic behaviours coincided with termite sickness, with a more rapid switch to cannibalism at later stages. By dividing the infection into stages^29^ and studying how the social immune response differs over time, our study sheds new light on the processes by which social Blattodea identify fatally ill colony members and thereby defend their colonies from disease.

## Results

### Patterns of behaviour

After exposing focal termites to *Metarhizium anisopliae* (*M.a*+) or a control Tween 80 solution (*M.a*−) and isolating them for 2, 12, 15, or 20 hours, we introduced them individually to groups of nestmates. These time points were chosen based on the results of a conidia germination experiment (Supplementary Material): at 2 hours, a *M. anisopliae*-exposed termite has conidia on its cuticle that have not yet begun to germinate; at 12 hours, germination has begun but the termite appears healthy; at 15 hours, the termite has become moribund; and at 20 hours, the *M. anisopliae*-exposed termite is near death. We observed the groups for three hours, scanning every 5 minutes and recording the state of the focal: groomed, cannibalised, buried, or “other” (states not related to social immunity, e.g. walking). Focal termites were visible throughout the three-hour observation period, and there were no instances of focal termite corpses being ignored.

**Figure 1:**
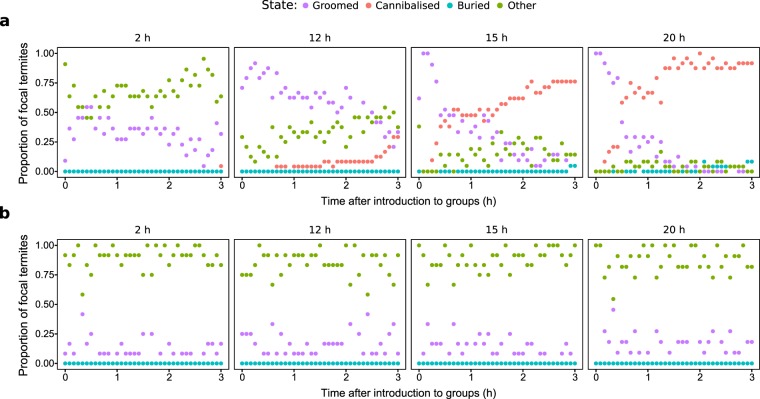
Patterns of behaviour over time during the three-hour observation period for (**a**) *M. anisopliae* and (**b**) control treatments. Each point represents the proportion of focal termites that were observed in a given state during that scan. When more than one state was present at the same proportion (0.50 or 0.00), the points overlap.

Behavioural patterns (Fig. 1) in the control treatments (2 h/*M.a*−, 12 h/*M.a*−, 15 h/*M.a*−, 20 h/*M.a*−) were broadly similar, dominated by states in the “other” category, with low levels of grooming and no cannibalism or burial states. The majority of states in 2 h/*M.a*+ were in the “other” category, but grooming was elevated over the control. 12 h/*M.a*+ was dominated by high levels of grooming that slowly decreased over the observation period, while “other” states slowly increased. Cannibalism was observed, but primarily in the last half hour of the observation period. No burial states were recorded. Both 15 h/*M.a*+ and 20 h/*M.a*+ were characterised by high levels of intense grooming immediately after the focal termites were introduced. Cannibalism began shortly thereafter, increasing more rapidly in 20 h/*M.a*+ and completely replacing grooming before the end of the observation period. Burial was observed in both treatments toward the end of the observation period, but only in a small proportion of states.

### Grooming

**Figure 2:**
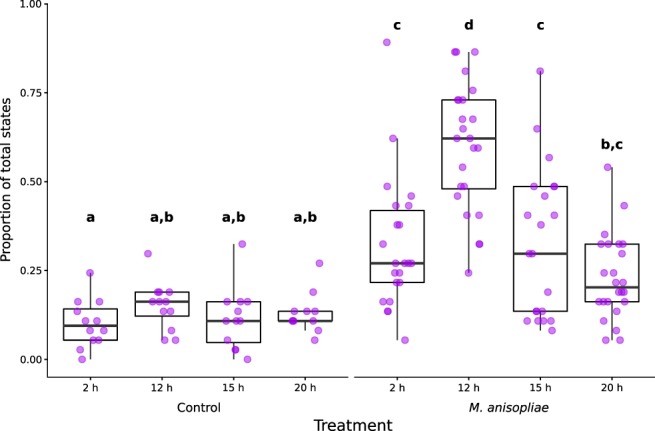
Grooming as a proportion of total states. Treatments marked with different letters were significantly different (Supplementary Table S1). Lower and upper hinges correspond to first and third quartiles, the upper whisker extends to the largest value if it is no greater than 1.5 times the inter-quartile rage from the hinge, and the lower whisker extends to the smallest value if it is no smaller than 1.5 times the inter-quartile range from the hinge.

The proportion of states classified as grooming in the 12 h/*M.a*+ treatment was significantly elevated over all other *M. anisopliae* treatments (12 h/*M.a*+ vs. 2 h/*M.a*+ z = 5.533 *P* < 0.001; 15 h/*M.a*+ vs. 12 h/*M.a*+ z = −5.511 *P* < 0.001; 20 h/*M.a*+ vs. 12 h/*M.a*+ z = −7.717 *P* < 0.001) (Fig. 2, Supplementary Table S1). Grooming was significantly elevated over the controls in all *M. anisopliae* treatments except 20 h/*M.a*+, which was only significantly different from 2 h/*M.a*− (*M. anisopliae* treatments vs. corresponding controls: 2 h/*M.a*+ vs. 2 h/*M.a*− z = 4.844 *P* < 0.001; 12 h/*M.a*+ vs. 12 h/*M.a*− z = 7.834 *P* < 0.001; 15 h/*M.a*+ vs. 15 h/*M.a*− z = 4.417 *P* < 0.001). The controls (2 h/*M.a*−, 12 h/*M.a*−, 15 h/*M.a*−, 20 h/*M.a*−) were not significantly different from each other. No significant difference was observed between 2 h/*M.a*+, 15 h/*M.a*+, and 20 h/*M.a*+ . Low levels of grooming in 15 h/*M.a*+ and 20 h/*M.a*+ correspond with a high proportion of cannibalism states in both treatments (Figs 1, 4).

**Figure 3:**
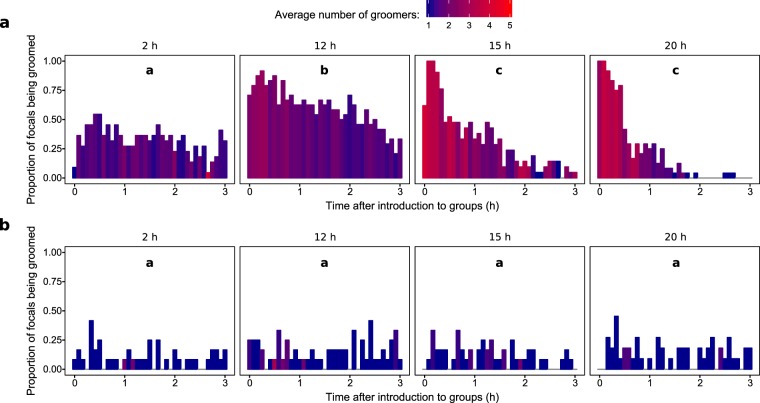
Proportion of focal termites in the (**a**) *M. anisopliae* or (**b**) control treatments observed being groomed by nestmates in each scan (as in Fig. 1), with the fill colour representing the average number of groomers involved. Different letters correspond to significant differences in the overall number of groomers after the number of grooming states is taken into account (Supplementary Table S2).

Only workers were observed grooming the focal termites, and grooming was visibly more intense, involving a significantly higher number of groomers, in 12 h/*M.a*+, 15 h/*M.a*+, and 20 h/*M.a*+ (12 h/*M.a*+ vs. 2 h/*M.a*+ z = 3.323 *P = *0.017; 15 h/*M.a*+ vs. 2 h/*M.a*+ z = 7.944 *P* < 0.001; 15 h/*M.a*+ vs. 12 h/*M.a*+ z = 5.777 *P* < 0.001; 20 h/*M.a*+ vs. 2 h/*M.a*+ z = 8.089 *P* < 0.001; 20 h/*M.a*+ vs. 12 h/*M.a*+ z = 5.867 *P* < 0.001) (Fig. 3, Supplementary Table S2). 2 h/*M.a*+ was not significantly different from any of the controls, and 15 h/*M.a*+ and 20 h/*M.a*+ *M.a*+ were not significantly different from each other. Non-focal termites in treatments with more intense grooming were frequently observed to engage in vibratory displays (jittering), a known pathogen alarm response^13,30^; however, our sampling method, which focused on direct interactions with the focal individual, precluded analysis of this behaviour.

### Cannibalism

**Figure 4:**
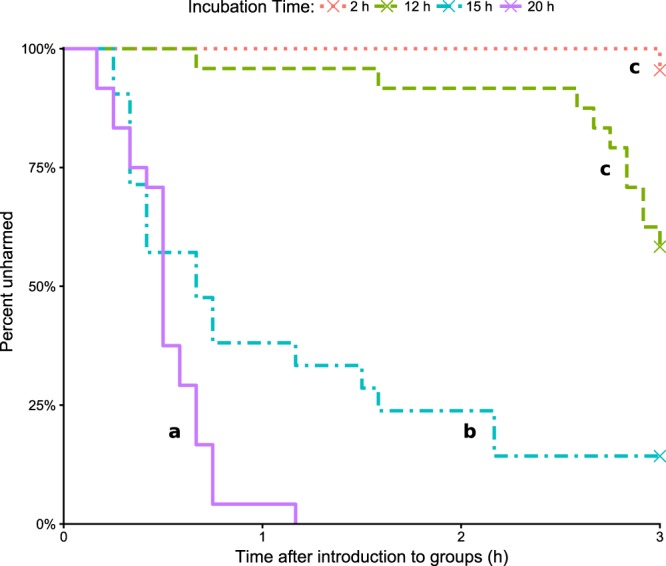
Percentage of *M. anisopliae*-treated focal termites that remained unharmed (not bitten or dismembered) over the three-hour observation period. X’s indicate the presence of right-censored data (i.e. focal termites that were not harmed during the observation period). Treatments marked with the same letter were not significantly different, and neither “c” treatment (2h/*M.a*+, 12h/*M.a*+) was significantly different from the controls (not shown; all control individuals remained unharmed throughout the observation period) (Supplementary Table S3).

The probability of remaining unharmed during the observation period was significantly different from the controls, which experienced no cannibalism, in the 15 h/*M.a*+ (z = 3.946 *P = *0.002) and 20 h/*M.a*+ (z = 4.845 *P* < 0.001) treatments, but not in 2 h/*M.a*+ or 12 h/*M.a*+ (Supplementary Table S3). The 15 h/*M.a*+ and 20 h/*M.a*+ treatments differed significantly from each other (z = 3.155, *P = *0.028), as well as from 2 h/*M.a*+ and 12 h/*M.a*+ (15 h/*M.a*+ vs. 2 h/*M.a*+ z = 4.542 *P* < 0.001; 15 h/*M.a*+ vs. 12 h/*M.a*+ z = 5.208 *P* < 0.001; 20 h/*M.a*+ vs. 2 h/*M.a*+ z = 5.504 *P* < 0.001; 20 h/*M.a*+ vs. 12 h/*M.a*+ z = 6.821 *P* < 0.001). This difference was characterised by a more rapid increase in cannibalism in 20 h/*M.a*+ than in 15 h/*M.a*+ (Fig. 4). In all but two cases, the first cannibalism-related state recorded was biting. In those two exceptions, both in 15 h/*M.a*+, it was dismemberment. The previous scans recorded intense grooming of the focal individual by five to six groomers: it is possible that those states were misidentified, or that biting began between scans. Cannibalism was performed primarily by workers, but on two occasions, a brachypterous neotenic was observed to also partake.

### Burial

Burial was observed in four plates: one 15 h/*M.a*+ replicate and three 20 h/*M.a*+ replicates. This is too few for meaningful statistical analysis. In each case, the focal termite appeared to be alive but moribund and largely immobile at the beginning of the burial process. This immobility was caused by nestmates in one 20 h/*M.a*+ replicate: the legs were first bitten off, and the maimed termite was left for approximately half an hour before burial began.

There was no sudden switch from grooming or cannibalism to burial. In one 20 h/*M.a*+ replicate, the focal termite was initially groomed, then bitten, then had a piece of paper placed on top of it (burial), then groomed again for half an hour, during which time the paper was removed, then bitten again. Burial did not resume, and the termite was eventually dismembered.

## Discussion

Our results demonstrate that *R. flavipes* colonies employ different collective immune defence strategies at different stages of infection with *M. anisopliae*. Before conidia germinate, the social immune response is dominated by grooming; however, contrary to our first hypothesis, levels of grooming rise significantly after conidial germination, and it becomes visibly more intense. Contrary to our second hypothesis, the onset of cannibalistic behaviour coincides with the stage of infection in which the termite becomes moribund, with a more rapid switch to cannibalism at later stages. All cannibalised individuals were eaten alive. This is consistent with observations by Rosengaus and Traniello^4^, who remarked that termites were usually cannibalised when near death, but contradicts Strack^24^, who observed more “agonism” toward healthy individuals that had been thickly dusted with conidia. Burial was rarely observed, reinforcing the view that termites preferentially eliminate sick individuals through cannibalism^4,23^.

The unexpectedly low levels of grooming observed before conidial germination may be explained by their weak attachment to the cuticle: since most conidia can be removed within hours by relatively few individuals^26^, there may be no reason to divert resources away from other colony functions or endanger additional members of the colony. The effectiveness of allogrooming, even at the observed low intensity, can also be seen in survivorship studies^14^. Increased levels of grooming after germination, then, could be linked to increased physical difficulty removing fungal material, especially after germ tube penetration.

This explanation is unsatisfying, because the longer a pathogen persists on or in members of a colony, the more we would expect it to affect colony fitness. Conidia-exposed, non-moribund individuals are mobile and can transfer conidia to many colony members^11,12^, all of which would need to be groomed by workers that could otherwise be performing other tasks. Should the infection progress to the next stage, the risk to the colony would increase significantly. This should favour early “clearance” of the infection from the colony via aggregation and intense grooming of conidia-exposed individuals, but that is not what we observed.

A second possibility is that the fungus-associated molecules that stimulate grooming (e.g. the fungal “odour”^31^) are partly masked, or present in lower quantities, before germination. Based on response threshold models of division of labour in social insects^32^, even partial masking would result in a weaker collective grooming response with fewer participating workers. This need not be a specific adaptation to evade termite social immunity, nor would we expect it in a generalist entomopathogen. Masking of immunogenic components of the fungal cell wall before (but not after) germination has previously been reported in an opportunistic human pathogen, *Aspergillus fumigatus* Fresenius^33^. Should this prove to be the case in *M. anisopliae*, it could be harnessed to develop strains with higher epizootic potential.

In contrast to grooming, in which fungal factors appear to be the primary trigger, the strong temporal correlation between moribundity and cannibalistic behaviour suggests that the host plays a central role in its own sacrifice. Focal termites appeared healthy at 12 hours and moribund (a reliable sign of internal infection^34^) at 15 h, and cannibalism was only prevalent in the 15 h/*M.a*+ and 20 h/*M.a*+ treatments. Even in the 12 h/*M.a*+ treatment, which was not significantly different from the control, there was an uptick in cannibalism in the last half hour of the observation period, i.e. at approximately 14.5 hours post-exposure. With the caveat that this is a correlation, and that moribundity could coincide with some fungus-derived stimulus reaching the necessary threshold for cannibalism, the hypothesis that sick individuals might flag themselves for destruction is supported by research in the social Hymenoptera. Ant pupae “advertise” the presence of an internal infection through modified cuticular hydrocarbon profile^3^, and aggressive behaviour was observed toward adults at the same stage of infection^10^; however, more work will be required to determine whether the social Blattodea and the social Hymenoptera have independently evolved separate mechanisms to identify fatally ill colony members, or if they have separately co-opted evolutionarily conserved sickness cues for social immune defence.

## Conclusion

We have demonstrated that termites can deploy different collective immune defences when confronted with a worker at different stages of infection with an entomopathogenic fungus. Whereas grooming is favoured earlier in the infectious process, moribund individuals are readily sacrificed. Cannibalism appears to be triggered by some factor associated with moribundity: what this might be remains unclear. Paradoxically, the termites did not display a robust social immune response at the earliest stages, when conidia had not yet germinated, although grooming was somewhat elevated. This may indicate that the ungerminated fungus is less visible to the “social immune system” of the colony, but this hypothesis remains to be tested.

This study adds to the body of knowledge surrounding termite social immunity and sheds light on how colonies resist fungal disease and regulate destructive immune behaviours. By dividing the infection into stages^29^ and studying how the social immune response differs over time, we can better understand how termites, and insects in general, defend their colonies from disease.

## Materials and Methods

### Insects

Three captive *Reticulitermes flavipes* colonies at the Federal Institute for Materials Research and Testing (Bundesanstalt für Materialforschung und -prüfung, BAM) in Berlin, Germany were used in these experiments: colonies E, 5, and 8. Colony E was collected in Soulac-sur-Mer, France, in 2015. It was maintained in a dark room at 28 °C, 83% humidity. Colonies 5 and 8 were collected in the vicinity of Le Grand-Village-Plage, Île d’Oléron, France, in 1994 and maintained in a separate dark room at 26 °C, 84% humidity. Colonies were housed in physically separate sheet metal tanks as described by Becker^35^. Each colony contained between ten and one hundred thousand individuals, which is typical for this species^36^. *Reticulitermes* spp. colonies are long-lived: primary reproductives can live some 18 years in the wild and 25 years in captivity^37^. *R. flavipes* colonies often have a large number of secondary reproductives which breed amongst themselves. This form of reproduction is known to be the norm in the French population of *R. flavipes* and is not uncommon in their native range^38^.

Cardboard bait was used as the primary method for extracting termites from their parent colonies. From collection until staining or transfer to Petri dish nests, termites from the same colony were maintained in a plastic box containing cellulose pads (Pall Corporation, Port Washington, USA) moistened with tap water. Each colony box was maintained under the same temperature and humidity conditions as the parent colony, and tap water and new cellulose pads were added as needed.

### Fungi

The entomopathogenic fungus *Metarhizium anisopliae* DSM 1490 was maintained on potato dextrose agar (PDA) at 25 °C in the dark. The plate used in the experiment was the result of one passage from a plate grown under identical conditions from a cryogenic stock.

### Experimental design

There are three key points in the fungal life cycle at which termites may detect a terminal infection. The earliest is after conidial germination: as a consequence of the thin, unsclerotised termite cuticle and the limited ability of the individual immune system to encapsulate germ tubes^34^, an internal infection can be established within hours of germination. In a conidia germination experiment, we observed germination on the termite cuticle as early as 10 hours after exposure, and visible signs of disease at 15 hours (Supplementary Material). The risks to the colony at this stage may outweigh the benefits of saving an individual on the cusp of infection. The second point is after internal infection has begun and the host has begun to show visible signs of disease^34^. Given the rapidity with which this fungus infects and kills its termite host^39^, it is possible that sickness cues such as volatiles or modifications to the cuticular hydrocarbon profile, if present, may take time to produce. The last point at which the colony might detect and respond to a terminal infection is therefore shortly before the termite’s death. In our conidia germination experiment, termites were alive but largely immobile at 20 hours after exposure, with death occurring at some point between 24 and 48 hours (Supplementary Material).

Based on these considerations, we chose to observe the collective responses to a worker termite at one of the following four stages of infection: (1) the conidia have attached but not germinated; (2) the conidia have begun to germinate but the host remains healthy; (3) the host is moribund (an internal infection has been established); and (4) the host is near death. To obtain individuals at each stage of infection, termites were treated with a 1 × 10^8^ conidia/mL suspension of *M. anisopliae* or 0.05% Tween 80 as a control, then maintained individually for 2, 12, 15, or 20 hours. These four incubation times correspond to the four stages of infection described above. Three colonies were used in the experiment. For each of the four incubation times, there were 24 replicates of the *M. anisopliae* treatment (eight per colony for three colonies) and 12 of the control treatment (four per colony) to control for the effects of handling and isolation. These were split evenly across two experimental replicates. In the first experimental replicate, the conidia used in the experiment were freshly harvested from half of one PDA plate. In the second experimental replicate, conidia were freshly harvested from the other half.

### Preparation of Petri dish nests

**Figure 5:**
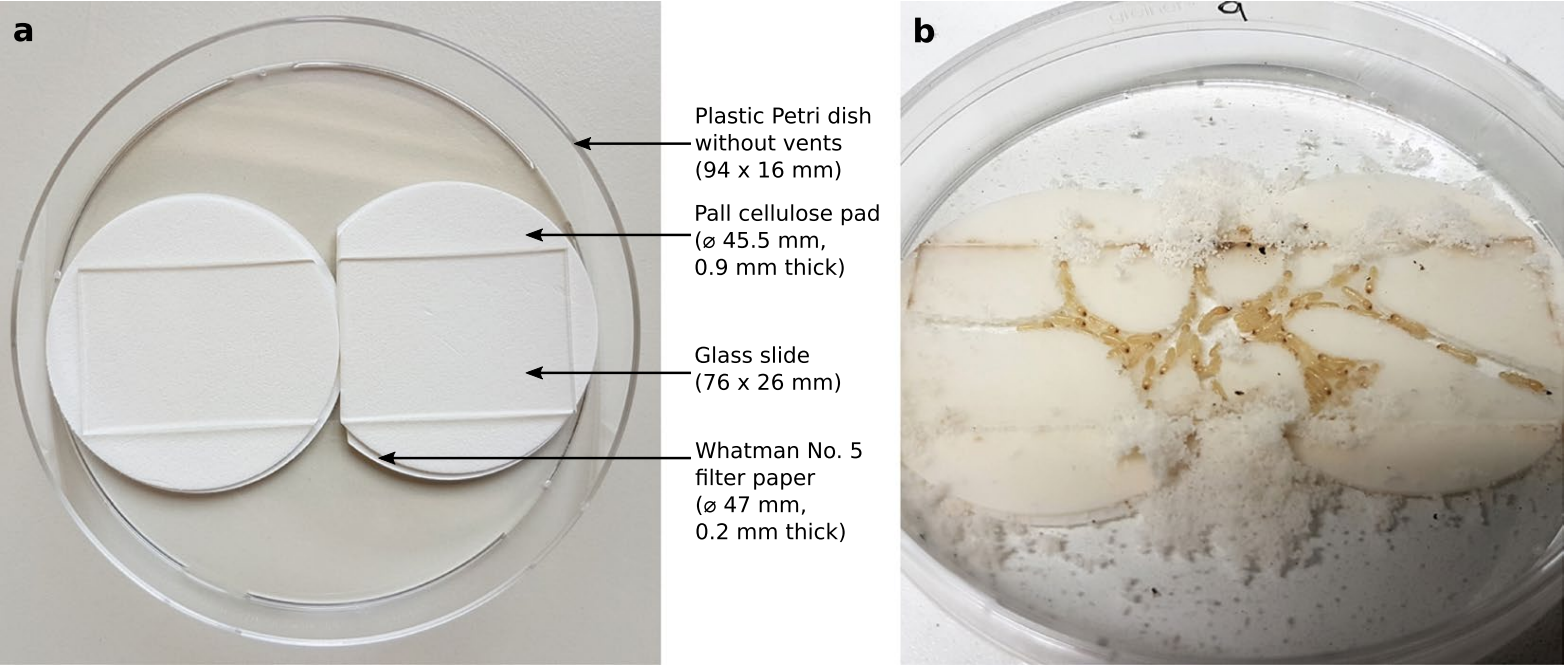
(**a**) Petri dish nest design; (**b**) Petri dish nest after two weeks of termite activity. Both photographs were taken by H. E. Davis.

Each Petri dish nest consisted of a Petri dish (94 × 16 mm, without vents), two thick Pall cellulose pads (45.5 mm diameter, 0.9 mm thick), two thin Whatman No. 5 filter paper discs (47 mm diameter, 0.2 mm thick), and one standard glass microscope slide (76 × 26 mm). Each thick cellulose pad was placed on top of a thin filter paper disc. The two paper stacks were then placed side-by-side in the Petri dish, with one of the stacks trimmed on one side to fit. Finally, a glass microscope slide was placed on top (Fig. 5). The thickness of the paper stacks (ca. 1.1 mm) was experimentally determined such that when termites dug in the paper under the glass slide, they were able to move freely but had too little space to leave an opaque “ceiling” over their tunnels. Immediately before the termites were added, the paper was moistened with tap water (3.5 mL). We did not add any water after this point.

Forty-five randomly selected medium-to-large workers were added to each Petri dish. Each dish also received three representatives of the reproductive caste, with the exception of seven plates from colony 5, which only received two in the second experimental replicate due to difficulty retrieving nymphs from the colony. We added one soldier to 20 dishes per colony (10 in the first experimental replicate, 10 in the second). It was not possible to retrieve enough soldiers to add one to every plate, and we were also unsure whether the presence of soldiers, which cannot participate in grooming, cannibalism, or burial, would influence pathogen response behaviour. *Reticulitermes flavipes* caste ratios vary^40^, but workers are consistently the dominant caste^18^. The numbers of reproductives and soldiers were taken into account in the statistical analysis, but neither had a significant effect.

In total, each dish contained 48 or 49 termites, not including the focal individual. Becker^35^ recommends using a minimum of 50 termites to maintain *R. flavipes* in the lab. In pilot experiments, we confirmed that groups this size could survive for three or more weeks in a Petri dish setup, and that they displayed typical social and hygienic behaviours, including cannibalism and burial. Smaller groups had lower survivorship and sometimes displayed abnormal behaviour, such as leaving corpses uneaten and unburied.

The dishes were sealed with parafilm to prevent desiccation and left in a dark room at 27 °C, 70% humidity for two weeks. At least half an hour prior to a behavioural experiment, a cotton swab was used to sweep debris off the glass slide. This was necessary to ensure a clear view into the nest.

### Marking focal termites

Focal termites were marked with Nile blue, a moderately toxic fat-soluble stain that has previously been used to mark termites in behavioural studies^17,18,41^. As an internal stain, it cannot be removed and does not interfere with grooming.

Our protocol is a faster version of Evans’ fast marking technique^42^. As termites will swallow any liquid that they are immersed in, we dispensed with his desiccation step. We used a concentration of 0.025% Nile blue as this was the minimum needed for reliable staining.

Large (≥4 mm) workers were poured into 2 mL microcentrifuge tubes, one per colony, using a small funnel. Only workers that appeared healthy and active were used. Sufficient 0.025% Nile blue was added to cover them, and they were flicked to mix for 1 minute, then tipped out onto a dry cellulose pad. Initially, all appeared unstained. The termites were transferred to one of three labelled round plastic containers (ca. 52 mm inner diameter), one per colony, each lined with a clean cellulose pad moistened with tap water (1 mL) and closed with a tight-fitting lid, and then left overnight in a dark room at 27 °C, 70% humidity. Only termites that were successfully stained and appeared healthy and active were used in the subsequent experiment. Because the intensity of the colour varied widely, and because of the known toxicity of the stain, termites of different shades were randomly distributed amongst treatments and controls.

### Preparation of conidial suspensions

Conidia were harvested after a minimum of one month of growth. A sterile cotton swab moistened with sterile 0.05% Tween 80 was used to wipe the conidia off the plate and suspend them in sterile 0.05% Tween 80. The suspension was inverted and vortexed to mix, then filtered through a piece of sterile cheese cloth that had been folded to reduce the effective pore size. The filtered conidia were washed by centrifuging for 10 minutes at 5000 g in a centrifuge cooled to 4 °C, discarding the supernatant, and resuspending the pellet in sterile 0.05% Tween 80. This step was performed a total of three times. Our harvest protocol was adapted from Yanagawa, A. *et al*.^17^.

A Thoma counting chamber (depth 0.1 mm) was used to estimate the concentration of the conidial suspension. Conidial suspensions were adjusted to 1 × 10^8^ conidia/mL with 0.05% Tween 80, aliquoted for ease of use, and used within 48 hours. Suspensions were stored at 4 °C when not in use.

To ensure that the conidia were viable, PDA plates were streaked with conidia from an aliquot of the same 1 × 10^8^ conidia/mL suspension used to inoculate the termites. The plates were parafilmed and placed upside-down in the same room as the termites (27 °C, 70% humidity). After 21 hours, at least 300 conidia were evaluated for germination at 200 to 400x magnification on one of the plates to calculate the germination rate. A conidium was considered germinated if the length of the germ tube was at least half the diameter of the conidium. For confirmation, at least 100 conidia were evaluated in the same manner on the second plate. The germination rate was ca. 94% in the first experimental replicate and ca. 98% in the second. A germination rate lower than 90% would have indicated a problem with the conidial suspension.

### Inoculation with conidia or 0.05% Tween 80

For the *M. anisopliae* treatment, previously-marked (blue) termites were placed in a round-bottomed 2 mL microcentrifuge tube, then covered with the 1 × 10^8^ conidia/mL suspension to a volume of 42 µL per termite. The tube was flicked to mix for 10 seconds, then poured out onto a dry cellulose pad. Termites that remained inside were tapped out, or, if needed, carefully removed with soft forceps. When the termites had recovered enough to walk, they were transferred one-by-one into separate Petri dishes, each containing a cellulose pad moistened with 1 mL tap water. The dishes were sealed with parafilm to prevent desiccation. Control termites were immersed in sterile 0.05% Tween 80 (42 µL per termite) instead of the conidial suspension and handled in the same way. This inoculation method is a variation on that used by Yanagawa and Shimizu^15^.

The *M. anisopliae*-treated and control termites were incubated for 2, 12, 15, or 20 hours at 27 °C, 70% humidity before use in the behavioural experiment.

### Behaviour recording

After 2, 12, 15, or 20 hours of incubation, the blue *M. anisopliae*-treated and control focal termites were added individually to the Petri dish nests. All dishes were resealed with parafilm. This took approximately 15 minutes, and the observation period began immediately after the last dish was sealed. Termites that appeared injured or dead at the beginning of an observation period were excluded from the analysis. In total, two replicates of the 2 h/*M.a*+ (2 hours of incubation with *M. anisopliae*) treatment, three replicates of the 15 h/*M.a*+ (15 hours of incubation with *M. anisopliae*) treatment, and one replicate of the 20 h/*M.a*+ (20 hours of incubation with *M. anisopliae*) treatment were excluded due to suspected handling injuries.

Scan sampling^43^ was used to observe the interactions between the focal termite and its nestmates within each Petri dish nest. Scans typically took less than 1 minute. They were performed every 5 minutes for a total of 3 hours using a magnifying glass (up to 3x magnification) to better distinguish between similar behaviours and a Samsung S7 smartphone as a digital voice recorder. All observations were made at 27 °C, 70% humidity under bright, constant overhead light. As *R. flavipes* are known to respond strongly to vibrational stimuli^44^, Petri dishes were not moved or opened after they had been sealed.

States were defined prior to the experiment. We classified behaviours into visually distinguishable, non-overlapping categories with a focus on interactions (and their aftermath) that are relevant to social immunity:

**Groomed by n**: Focal termite is being groomed by n nestmates with no evidence of biting.

**Bitten**: Focal termite is being bitten by one or more nestmates.

**Dismembered**: Focal termite is missing one or more tagmata.

**Dead-ignored**: Focal termite is lying completely motionless, but not buried or dismembered. Nestmates are not interacting with it.

**Not visible**: Focal termite is in a section of the nest where its behaviour and interactions with nestmates cannot be seen.

**Other**: Focal termite is alive, intact, and unburied, but nestmates are not interacting with it.

### Statistical analysis

All statistical analyses were performed using R version 3.4.3^45^.

### Grooming

The amount of grooming in each treatment (number of grooming states/total observed states) was compared by fitting a generalised linear mixed model to the data using the glmer function in the package lme4^46^. Because we were working with proportion data, we used a binomial error structure^47^.

The model contained an interaction between incubation time and *M. anisopliae* presence as a fixed effect and three random effects: (1) colony, (2) experimental replicate, and (3) Petri dish nest ID. We initially included soldier number and reproductive number as fixed effects, then sequentially removed them during model simplification, using the anova function to ascertain if the removal of a parameter would lead to a significant change in deviance and to perform likelihood ratio test comparisons. The final model was tested for overdispersion using the dispersion_glmer function in the package blmeco^48^. A scale parameter between 0.75 and 1.4 indicates no overdispersion: for this model, it was 1.004. All post hoc pairwise comparisons were performed using the glht function from the multcomp package^49^ with Tukey correction.

To analyse grooming intensity, we used glmer to fit a generalised linear mixed model to the data with the total number of groomers in each replicate as the response variable and a Poisson error structure. Two replicates (one in the 2 hour control treatment, one in the 15 hour control treatment) were excluded from the analysis because no grooming states were observed. The model contained an interaction between incubation time and *M. anisopliae* presence as a fixed effect and three random effects: (1) colony, (2) experimental replicate, and (3) Petri dish nest ID. Because each observed grooming state increased the number of groomers by at least one, and the number of grooming states varied between replicates, the log of the number of grooming states was used as an offset. We initially included soldier number and reproductive number as fixed effects, then sequentially removed them during model simplification, using anova as above to compare models. The scale parameter of the final model was 0.859. All post hoc pairwise comparisons were performed using glht with Tukey correction.

### Cannibalism

“Bitten” and “dismembered” states were combined into a single “cannibalism” state. We modelled the onset of cannibalistic behaviour using survival curves. The data were plotted using survfit from the survival package^50,51^ and ggsurvplot from the survminer package^52^. We used a mixed effects Cox model (coxme from the coxme package^53^) to compare the curves.

The model contained an interaction between incubation time and *M. anisopliae* presence as a fixed effect, and colony and experimental replicate as random effects. We initially included soldier number and reproductive number as fixed effects, then sequentially removed them during model simplification, using the anova function as above to compare models. In the survival curve analysis, all control data was initially right-censored; in order to fit a mixed effects Cox model to the data, it was necessary to uncensor one arbitrarily-selected control replicate from each incubation time treatment following Tragust, Ugelvig, Chapuisat, Heinze, and Cremer^54^. The glht function was used to perform post-hoc pairwise comparisons with Tukey correction.

## Electronic supplementary material

Supplementary Information

Supplementary Dataset 1

## Data Availability

All data generated or analysed during this study are included in this published article (and its Supplementary Information files).
